# Applicability of two commonly used bone age assessment methods to twenty-first century UK children

**DOI:** 10.1007/s00330-019-06300-x

**Published:** 2019-08-01

**Authors:** Khalaf Alshamrani, Amaka C. Offiah

**Affiliations:** 1 Department of Oncology & Metabolism, University of Sheffield, Sheffield, UK; 2 College of Applied Medical Sciences, Najran University, Najran, Saudi Arabia; 3 Academic Unit of Child Health, Sheffield Children’s NHS Foundation Trust, Damer Street Building, Western Bank, Sheffield, S10 2TH UK; 4 Sheffield Children’s NHS Foundation Trust, Western Bank, Sheffield, UK

**Keywords:** Age determination by skeleton, Forensic medicine, X-rays, Hand, Wrist

## Abstract

**Objectives:**

To assess the effect of secular change on skeletal maturation and thus on the applicability of the Greulich and Pyle (G&P) and Tanner and Whitehouse (TW3) methods.

**Methods:**

BoneXpert was used to assess bone age from 392 hand trauma radiographs (206 males, 257 left). The paired sample *t* test was performed to assess the difference between mean bone age (BA) and mean chronological age (CA). ANOVA was used to assess the differences between groups based on socioeconomic status (taken from the Index of Multiple Deprivation).

**Results:**

CA ranged from 2 to 15 years for females and 2.5 to 15 years for males. Numbers of children living in low, average and high socioeconomic areas were 216 (55%), 74 (19%) and 102 (26%) respectively. We found no statistically significant difference between BA and CA when using G&P. However, using TW3, CA was underestimated in females beyond the age of 3 years, with significant differences between BA and CA (− 0.43 years, SD 1.05, *p* = < 0.001) but not in males (0.01 years, SD 0.97, *p* = 0.76). Of the difference in females, 17.8% was accounted for by socioeconomic status.

**Conclusion:**

No significant difference exists between BoneXpert-derived BA and CA when using the G&P atlas in our study population. There was a statistically significant underestimation of BoneXpert-derived BA compared with CA in females when using TW3, particularly in those from low and average socioeconomic backgrounds. Secular change has not led to significant advancement in skeletal maturation within our study population.

**Key Points:**

*• The Greulich and Pyle method can be applied to the present-day United Kingdom (UK) population.*

*• The Tanner and Whitehouse (TW3) method consistently underestimates the age of twenty-first century UK females by an average of 5 months.*

*• Secular change has not advanced skeletal maturity of present-day UK children compared with those of the mid-twentieth century.*

**Electronic supplementary material:**

The online version of this article (10.1007/s00330-019-06300-x) contains supplementary material, which is available to authorized users.

## Introduction

Bone age assessment plays an important role in clinical practice, permitting investigation of whether bone maturity is occurring at a rate consistent with chronological age (CA). In this context, bone age (BA) assessment is useful for managing children with skeletal dysplasias and endocrine disorders, as well as planning for orthopaedic procedures [1]. Approximately 160,000 unaccompanied children entered European countries during 2015 and 2016 [2]. Although there is no precise figure, numbers are significant and authorities have faced challenges in estimating some of their ages [3]. In these situations, CA has occasionally been deduced by comparing BA of the individual in question with the existing BA standards [4]. This practice is particularly common at geographical borders where conflicts or crises are occurring. Whether to aid clinical management of paediatric patients or to determine chronological age when this is unknown, it is crucial to have a reliable and appropriate method of determining bone age [5]. However, the European Society of Paediatric Radiology musculoskeletal task force has recently advised against the practice of estimating chronological age based on an assessment of bone age [6].

Numerous approaches have been developed to determine BA. Among these, two methods are widely utilised based on left hand and wrist radiographs, namely the Greulich and Pyle (G&P) and Tanner and Whitehouse (TW) methods [7, 8]. The G&P method is based on matching the child’s hand radiograph to standard plates provided by the G&P atlas; thus, this method compares the hand’s general maturational status. The population providing the G&P standard atlas were originally North American Caucasians of “good” socioeconomic status in 1938. The “good” socioeconomic status was designated because recruited children were above average both economically and educationally (they were also free of physical, mental, nutritional and environmental factors detrimental to growth) [9]. In contrast to the G&P atlas, the TW method undertakes an assessment and scoring of skeletal maturity for each individual hand and wrist bone. Data provided by the Harpenden Longitudinal Growth Study enabled the TW method’s development. In 2001, the TW3 method replaced the TW1 and TW2 methods as a result of documented secular change (as stated by the authors). The data that formed the TW3 method was collected from European and American Caucasian children of average socioeconomic status during the 1980s and 1990s [10]. Following the introduction of G&P and TW3 standards, numerous investigations have been undertaken internationally, in order to identify the extent to which these standards are relevant to various populations. This issue is significant, especially in light of the growing volume of studies concluding that certain methods are inappropriate for particular ethnic groups and as a result of improvements in socioeconomic status [11–14].

BoneXpert software was developed in 2009, enabling automatic calculation of bone age, according to the G&P and TW3 standards [15]. The software provides standard deviation scores for each hand radiograph, thus assisting the comparison of a child’s bone age with healthy children of the same sex and age. There are several advantages in utilising this software tool, including eliminating observer variability and saving rating times.

This study aims to use BoneXpert to test the applicability of the G&P and TW3 methods to United Kingdom (UK) children born in the twenty-first century, whose standard of living (across all socioeconomic categories) is likely to be higher than that of the children used to develop the G&P and TW3 methods. Our hypothesis was that improved living standards and therefore improved nutrition would render their bone age advanced when compared with their chronological age [16].

## Methods

### Study design

Hand radiographs performed between 2010 and 2016 on children aged between 2 and 15 years presenting to the Emergency Department of Sheffield Children’s Hospital, United Kingdom, following upper limb trauma, were retrospectively identified from the Picture Archiving and Communication System.

Radiographs that contained recent untreated fractures were used. However, radiographs in children with a history of previous fracture were excluded, as were those with a specific request for BA estimation. When both the left and right hands were imaged in the same child, only the left hand radiograph was included in the analysis. Demographic data including sex, ethnicity (self-reported) and CA at the time of the radiograph were recorded.

Socioeconomic status of recruited children was documented using the Index of Multiple Deprivation (IMD) [17]. The postcode of each child was retrieved from the patient address data and then the corresponding values provided by the IMD for each postcode were recorded. The IMD measures deprivation based on income, employment, education, health and disability, crime, barriers to housing and service and living environment for each small area. These small areas consist on average of 650 households and approximately 1500 residents [18]. The English IMD 2015 data are ranked for each small area within England from 1 to 32,844. IMD scores below 10,894 are deemed to be areas of low socioeconomic status, between 10,895 and 21,788 are average, and above 21,789 are of high socioeconomic status. BoneXpert software (Visiana) was utilised to analyse the hand radiographs. All radiographs were acquired via a computed radiography system and were in DICOM format. The default ethnicity for analysing the radiographs was Caucasian, because the software does not include ethnicity-specific standard deviation scores (SDS).

### Statistical analysis

Statistical analysis was undertaken via SPSS version 24 for PC (IBM). The mean variation for BA and CA was determined for each child by subtracting BA from CA (BA − CA). Therefore, a positive value indicates advanced BA, whereas a negative value indicates delayed BA, compared with CA. The significance of the differences was calculated using a paired sample *t* test.

Statistical analysis was undertaken separately for both sexes, in relation to each method (G&P and TW3) and the standard error of the estimate (SEE) was calculated for each sex and method (all ethnicities) [19]. Analysis was repeated for both sexes for Caucasians only, to investigate the effect of ethnicity on the results. Analysis was also performed to determine the effect of readings from left and right hands. The effect of socioeconomic status was evaluated using the one-way ANOVA test. Results were considered statistically significant when the *p* value was < 0.05 (two-sided).

Approval was obtained from the Health Research Authority at Yorkshire and Humber. The need for full Research Ethics Committee approval was waived for this retrospective study of hand radiographs.

## Results

**Fig. 1:**
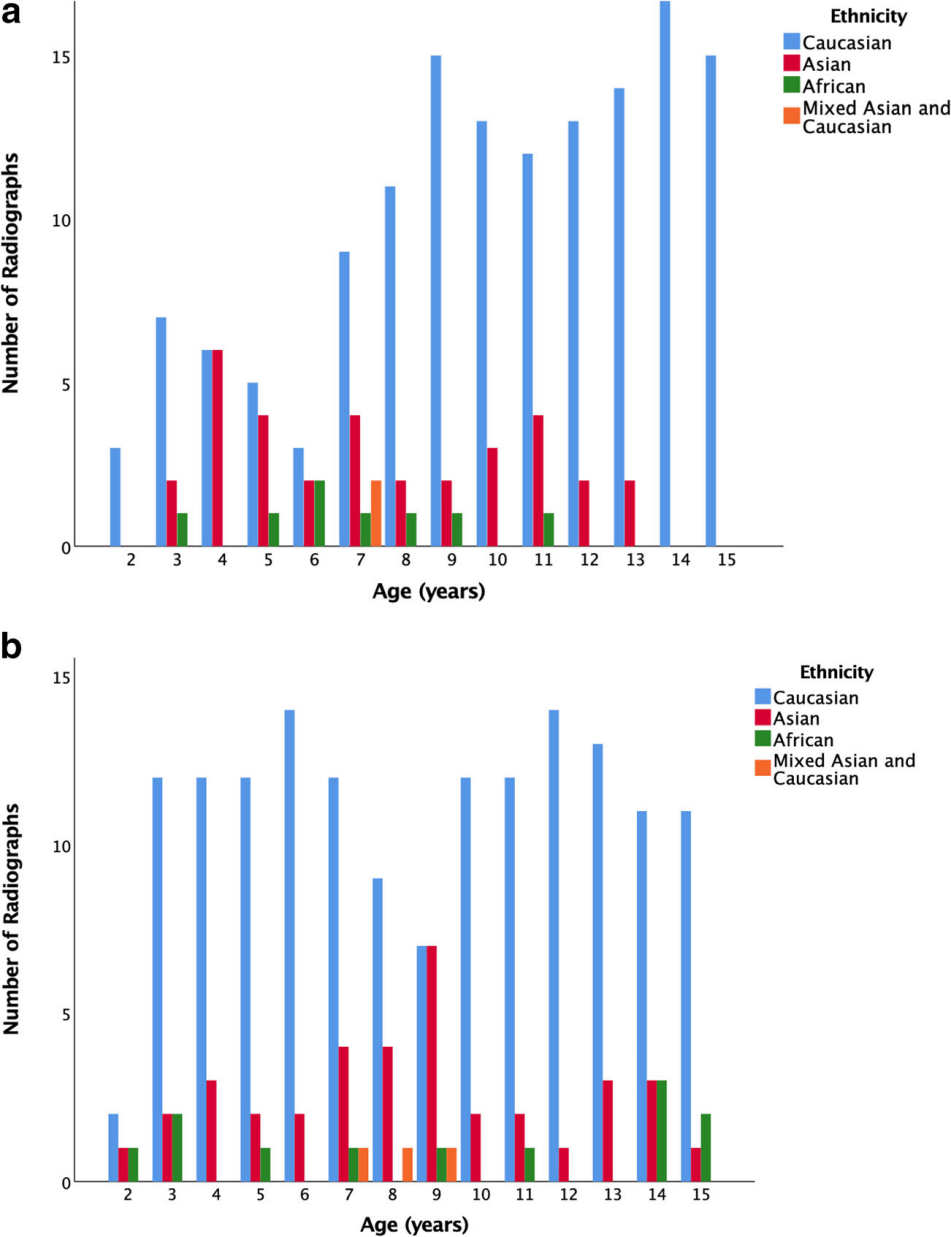
Number of included children by age and ethnic group. **a** Females. **b** Males

In total, we identified 401 potentially eligible hand and wrist radiographs of which 9 were omitted due to BoneXpert failing to provide a reading for the following reasons as provided by the software: (1) “radiograph too sharp” in six images (this terminology is provided by the software for images with excessive edge enhancement or other post-processing), (2) poor image quality in two and (3) inconsistent lengths in one. Therefore, results are from 392 radiographs, comprising 206 males, 296 Caucasians, 71 Asians, 20 Africans and 5 mixed (Caucasian/Asian). Figure 1 illustrates the number of children per age and sex. In regard to socioeconomic status, 216 (55%), 74 (19%) and 102 (26%) children were of low, average and high socioeconomic status, respectively.

Concerning G&P, mean difference between BA and CA ranged from 33-month underestimation to 36-month overestimation in both females and males. Although differences were not statistically significant, G&P underestimated females’ ages by 1 month and overestimated males’ ages by 1.6 months (Table 1). BA was lower than CA in 51% of females and 44% of males, while being equal in 1% of males. With the cohort divided into yearly intervals, G&P overestimated females aged from 2 to 7 years by between 0.8 and 6 months, apart from at 4 years of age. This overestimation was statistically significant (*p* < 0.05) at age 6, in females (Table 2). After 7 years of age, G&P consistently underestimated females until 12 years of age by between 0.1 and 11 months, with underestimation being statistically significant (*p* < 0.05) at 12 years of age (Table 2). Subsequently, G&P overestimated females’ ages. Concerning males, G&P overestimated in all age groups apart from at 3, 6 and 12 years of age, with no statistical difference between BA and CA. ANOVA test showed no statistical difference between low, average and high socioeconomic status groups when using the G&P atlas for either females (*p* = 0.171) or males (*p* = 0.204). However, in females, the mean difference between BA and CA tended to be larger in low and average socioeconomic status groups, while in males, the difference tended to be larger within the higher socioeconomic status group.

**Table 1:** Mean difference (SD) in years, between BA and CA in females and males

	Sex	Mean CA (SD)	Mean BA (SD)	Mean difference BA − CA	*p* value
All ethnicities					
G&P BA vs CA	Female	9.96 (3.78)	9.89 (3.84)	− 0.07 (1.05)	0.326
	Male	9.32 (3.91)	9.45 (4.06)	0.13 (1.01)	0.063
TW3 BA vs CA	Female	9.96 (3.78)	9.53 (3.54)	− 0.43 (1.12)	< 0.001
	Male	9.32 (3.91)	9.34 (3.71)	0.02 (0.92)	0.764
Caucasians only					
G&P BA vs CA	Female	10.57 (3.62)	10.45 (3.81)	− 0.12 (1.06)	0.176
	Male	9.44 (3.85)	9.46 (4.10)	0.02 (1.05)	0.793
TW3 BA vs CA	Female	10.57 (3.62)	10.03 (3.54)	− 0.54 (0.96)	< 0.001
	Male	9.44 (3.85)	9.31 (3.82)	− 0.13 (0.64)	0.091

**Table 2:** Mean difference (SD) in years, between G&P BA and CA (all ethnicities)

	Age (years)	All ethnicities			Caucasians only		
		Mean	SD	*p* value	Mean	SD	*p* value
Males	2	0.07	0.43	0.784	0.19	0.09	0.202
	3	− 0.08	0.96	0.747	− 0.41	0.75	0.083
	4	0.01	0.90	0.962	− 0.14	0.95	0.614
	5	0.00	1.10	0.989	− 0.11	0.98	0.692
	6	− 0.13	0.80	0.530	− 0.28	0.70	0.158
	7	0.24	1.05	0.346	0.11	0.97	0.682
	8	0.43	1.29	0.231	0.16	1.27	0.713
	9	0.49	1.23	0.132	0.65	1.46	0.285
	10	0.33	1.00	0.240	0.32	1.09	0.314
	11	0.34	1.13	0.260	0.09	1.09	0.761
	12	− 0.13	1.00	0.612	− 0.17	1.02	0.520
	13	0.14	1.09	0.620	− 0.11	0.99	0.680
	14	0.02	1.06	0.953	0.22	1.05	0.786
	15	0.20	1.52	0.632	0.35	1.56	0.461
Females	2	0.11	0.07	0.121	0.10	0.07	0.126
	3	0.35	0.73	0.168	0.56	0.69	0.078
	4	− 0.21	0.96	0.468	− 0.1	0.75	0.578
	5	0.12	0.95	0.710	0.1	0.78	0.975
	6	0.50	0.39	0.015	0.69	0.34	0.072
	7	0.07	0.76	0.725	− 0.29	0.50	0.123
	8	− 0.46	1.06	0.130	− 0.65	0.83	0.021
	9	− 0.01	0.95	0.975	0.04	0.98	0.869
	10	− 0.13	1.18	0.659	− 0.19	1.24	0.582
	11	− 0.47	1.13	0.107	− 0.49	1.05	0.124
	12	− 0.94	0.99	0.002	− 1.06	0.7	0.001
	13	0.12	1.11	0.673	0.1	1.17	0.756
	14	0.49	1.45	0.187	0.48	1.45	0.185
	15	− 0.05	0.87	0.822	− 0.51	0.86	0.822

**Fig. 2:**
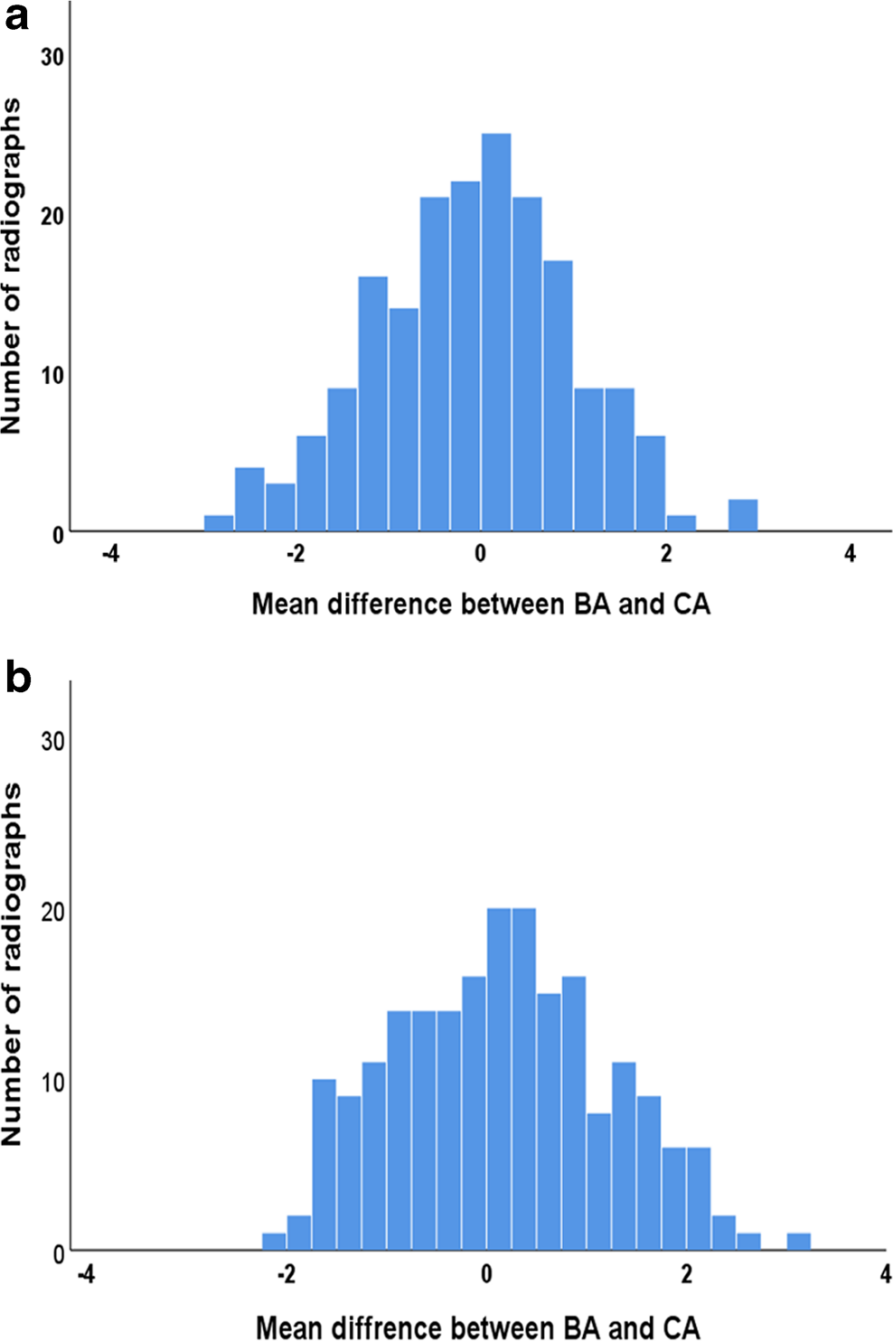
Distribution of mean difference between G&P BA and CA (in years). **a** Females. **b** Males

**Fig. 3:**
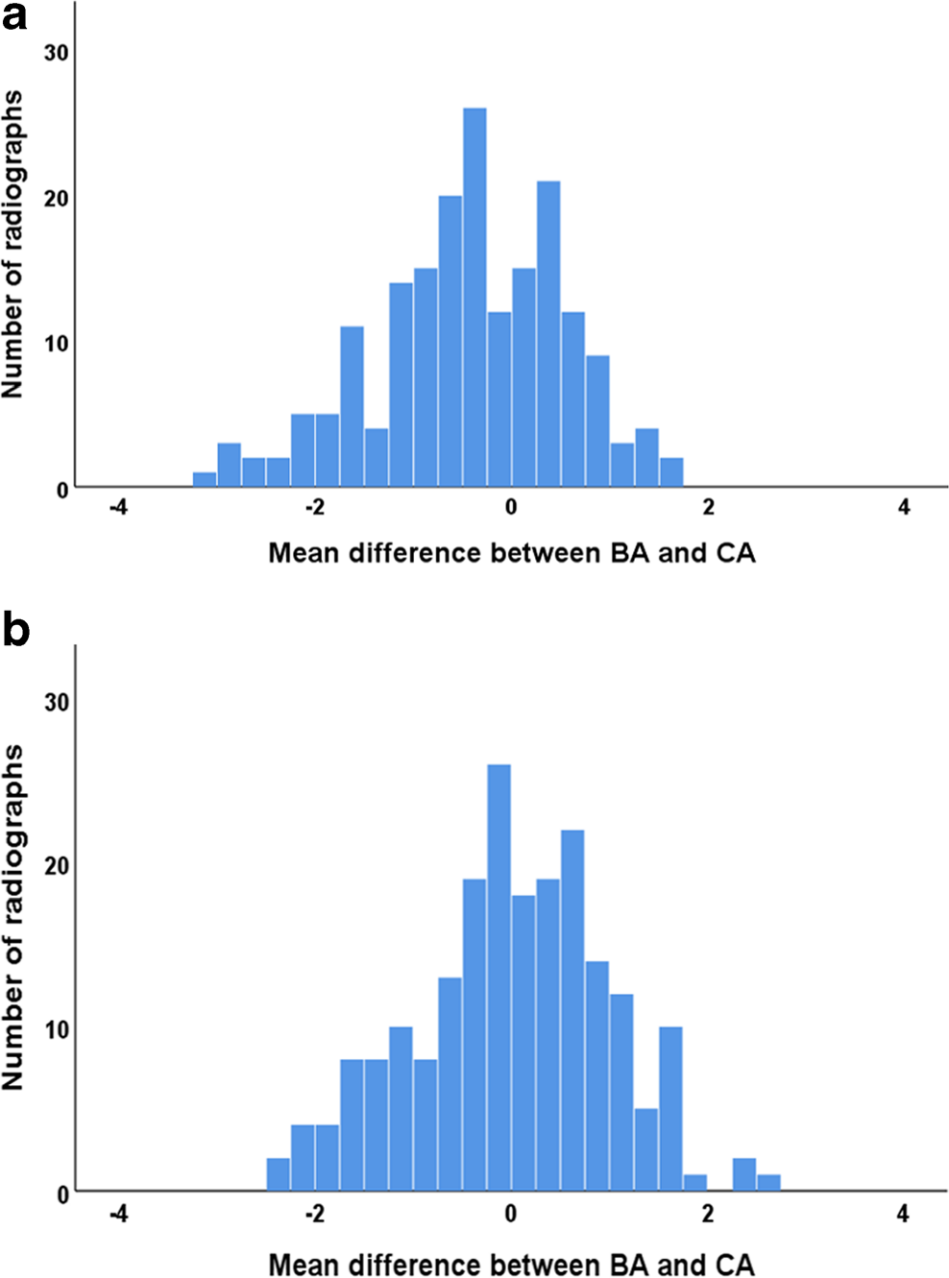
Distribution of mean difference between TW3 BA and CA (in years). **a** Females. **b** Males

Concerning TW3, overall mean difference between BA and CA showed a statistically significant difference in females but not in males. The mean difference between BA and CA ranged from 37-month underestimation to 32-month overestimation in both females and males. BA was lower than CA in 64.5% of females and 49.5% of males, while being equal in 0.5% of males. TW3 underestimated females’ ages by between 2 and 15 months (mean 5.2 months, *p* < 0.01) for all chronological age groups above 3 years (Table 3). TW3 significantly underestimated females at 8, 11, 12 and 15 years of age (*p* < 0.05). There was a statistically significant difference between the three socioeconomic groups as determined by one-way ANOVA (*p* = 0.019). Post hoc ANOVA showed that 17.8% of the variation between CA and TW3. BA as assessed by BoneXpert was accounted for by socioeconomic status. Observed differences were larger and significant (*p* < 0.001) in females of low and average socioeconomic status (Table 4). In males, TW3 underestimated age for those 10 years or above; this was statistically significant in Caucasians at ages 9, 12 and 13 years. There was no statistically significant difference between socioeconomic groups as determined by one-way ANOVA (*p* = 0.91). Distribution of the mean difference between CA and BA estimated via both G&P and TW3 methods for each sex is illustrated in Figs. 2 and 3.

**Table 3:** Mean difference (SD) in years, between TW3 BA and CA (all ethnicities)

	Age (years)	All ethnicities			Caucasians only		
		Mean	SD	*p* value	Mean	SD	*p* value
Males	2	0.61	0.29	0.022	–	–	–
	3	0.34	0.76	0.083	0.08	0.73	0.748
	4	0.11	0.76	0.592	− 0.18	0.81	0.514
	5	0.10	1.02	0.695	− 0.6	1.14	0.883
	6	− 0.08	0.96	0.759	− 0.2	0.95	0.328
	7	0.40	1.02	0.114	0.33	0.80	0.305
	8	0.36	0.98	0.180	0.10	1.06	0.799
	9	0.23	1.00	0.384	0.76	0.75	0.058
	10	− 0.07	0.76	0.735	0.05	0.84	0.875
	11	− 0.12	1.05	0.673	− 0.47	1.04	0.231
	12	− 0.50	1.07	0.097	− 0.68	1.03	0.057
	13	− 0.23	1.08	0.406	− 0.9	0.70	< 0.001
	14	− 0.32	1.03	0.212	− 0.33	1.22	0.553
	15	− 0.45	1.09	0.144	− 0.33	1.21	0.432
Females	2	0.34	0.19	0.097	0.33	0.19	0.092
	3	0.44	0.45	0.014	0.73	0.30	0.017
	4	− 0.21	0.58	0.234	− 0.13	0.50	0.583
	5	− 0.26	0.74	0.297	− 0.10	0.58	0.731
	6	− 0.18	0.46	0.331	0.07	0.56	0.855
	7	− 0.29	0.78	0.159	− 0.7	0.56	0.019
	8	− 0.75	1.15	0.035	− 0.61	0.60	0.034
	9	− 0.24	0.95	0.302	− 0.32	1.13	0.393
	10	− 0.38	1.14	0.190	− 0.21	1.21	0.621
	11	− 0.72	1.03	0.011	− 0.76	1.13	0.093
	12	− 1.28	0.93	< 0.001	− 1.69	0.36	< 0.001
	13	− 0.27	1.28	0.408	− 0.47	0.73	0.142
	14	− 0.33	1.04	0.216	− 0.28	1.01	0.388
	15	− 0.88	0.32	< 0.001	− 0.87	0.19	< 0.001

**Table 4:** Mean difference (SD) in years, between G&P, TW3 and CA in three socioeconomic groups

		*n*	Females	Males
Mean difference between BA and CA (SD) G&P–CA				
All ethnicities	Low	213	− 0.23 (1.11)	0.10 (1.12)
	Average	75	− 0.35 (1.03)	0.14 (0.97)
	High	101	0.06 (1.0)	0.26 (1.05)
Caucasians	Low	149	− 0.19 (1.02)	− 0.04 (1.10)
	Average	59	− 0.33 (1.01)	0.08 (0.88)
	High	86	− 0.02 (1.12)	0.14 (1.08)
TW3–CA				
All ethnicities	Low	213	− 0.52 (0.86)*	− 0.01 (1.03)
	Average	75	− 0.63 (0.98)*	− 0.02 (0.79)
	High	101	− 0.37 (0.87)*	0.06 (0.92)
Caucasians	Low	149	− 0.58 (0.94)*	− 0.24 (0.97)
	Average	59	− 0.66 (0.96)*	− 0.7 (0.86)
	High	86	− 0.47 (0.93)*	− 0.2 (0.91)

*p value < 0.01

Analysis of the Caucasian data showed no statistically significant difference when compared with the results from overall analysis, which included all ethnicities (Tables 1 and 2). In particular, the mean difference between CA and BA estimated by TW3 was statistically significant both in females of all ethnicities and in Caucasian females alone. An independent *t* test showed no significant difference between the mean difference of BA and CA when acquired from either the left hand or the right hand for both G&P (*p* = 0.58 females, *p* = 0.07 males) and TW3 (*p* = 0.08 females, *p* = 0.30 males) methods. Mean differences between BA and CA according to body side are illustrated in Table 5.

**Table 5:** Mean difference between BA and CA in years, according to body side (all ethnicities)

		Females		Males	
		Left hand *n* = 118	Right hand *n* = 68	Left hand *n* = 139	Right hand *n* = 67
G&P	Mean difference (SD)	0.03 (1.06)	− 0.2 (1.02)	0.1 (1.08)	0.21 (1.03)
TW3	Mean difference (SD)	− 0.32 (0.94)	− 0.6 (0.99)	− 0.04 (1.00)	0.09 (0.95)

The G&P and TW3 methods showed comparable accuracy in females with the standard error of the estimate (SEE) of ± 1.05 and ± 1.06 years, respectively. Similar accuracy for the two methods was also observed in males with SEE of ± 1.10 and ± 1.00 years for G&P and TW3 respectively.

## Discussion

Several variables may affect the applicability of BA methods. One is socioeconomic status, which refers to a combination of environmental factors such as nutritional status, state of health and economical and social class of an individual. Being of “high” socioeconomic status infers improved access to healthcare, sufficient food, exercise and housing, allowing full growth potential to be achieved [20]. Studies have shown that high socioeconomic status is more likely to accelerate skeletal maturation rate [12]. This might be related to nutritional factors with over-nutrition leading to overweight/obesity, which in children has been linked to BA advancement [21, 22]. In contrast, individuals from low socioeconomic groups are more likely to have poor diets and lower weight and are more likely to experience growth retardation [23]. Bearing in mind that the TW2 method was updated because of perceived effects of secular change [8], whereas G&P has never been updated, we questioned the reliability of bone age assessment methods. We sought to analyse the reliability of the G&P and TW3 methods within the modern-day UK context.

Breaking the cohort into yearly intervals showed statistical significance for varying age groups in females and males, when using the G&P atlas. These differences (overestimation at age of 6 and underestimation at age of 12, in females) were still significant when only data from Caucasian children was analysed. In spite of these sub-group differences, there was no statistical difference between overall mean BA and overall mean CA in either males or females. To convey a comprehensive picture, we contrasted our findings—especially mean difference between BA and CA—with previous studies that focused on the Caucasian population (Supplementary Table 1). Some of these studies have concluded that Caucasian children mature skeletally at approximately the same rate as the G&P standard in males across all age groups [14, 24–28]. However, other authors recommend that the G&P atlas be used with reservation due to mean BA being retarded in some age groups compared to the reference population [29–32]. Common findings among these studies of the G&P atlas include underestimation of males aged below 13 years and overestimation during adolescence [30–36]. G&P was applicable to females during adolescence while overestimation was reported before the age of 12 years [31, 32]. Others have recommended that a new standard altogether is required for precise bone age assessment, given the significant advancement of BA due to secular changes in skeletal maturation, which is thought to be due to improved standard of living [28, 30, 35, 36]. For example, Calfee et al reported that G&P overestimated males and females between 12 and 15 years old, for whom BA exceeded CA by at least 2 years [35]. All of these studies used the subjective assessment of experienced raters; our results using an objective software program indicate that overall, G&P currently remains applicable.

**Fig. 4:**
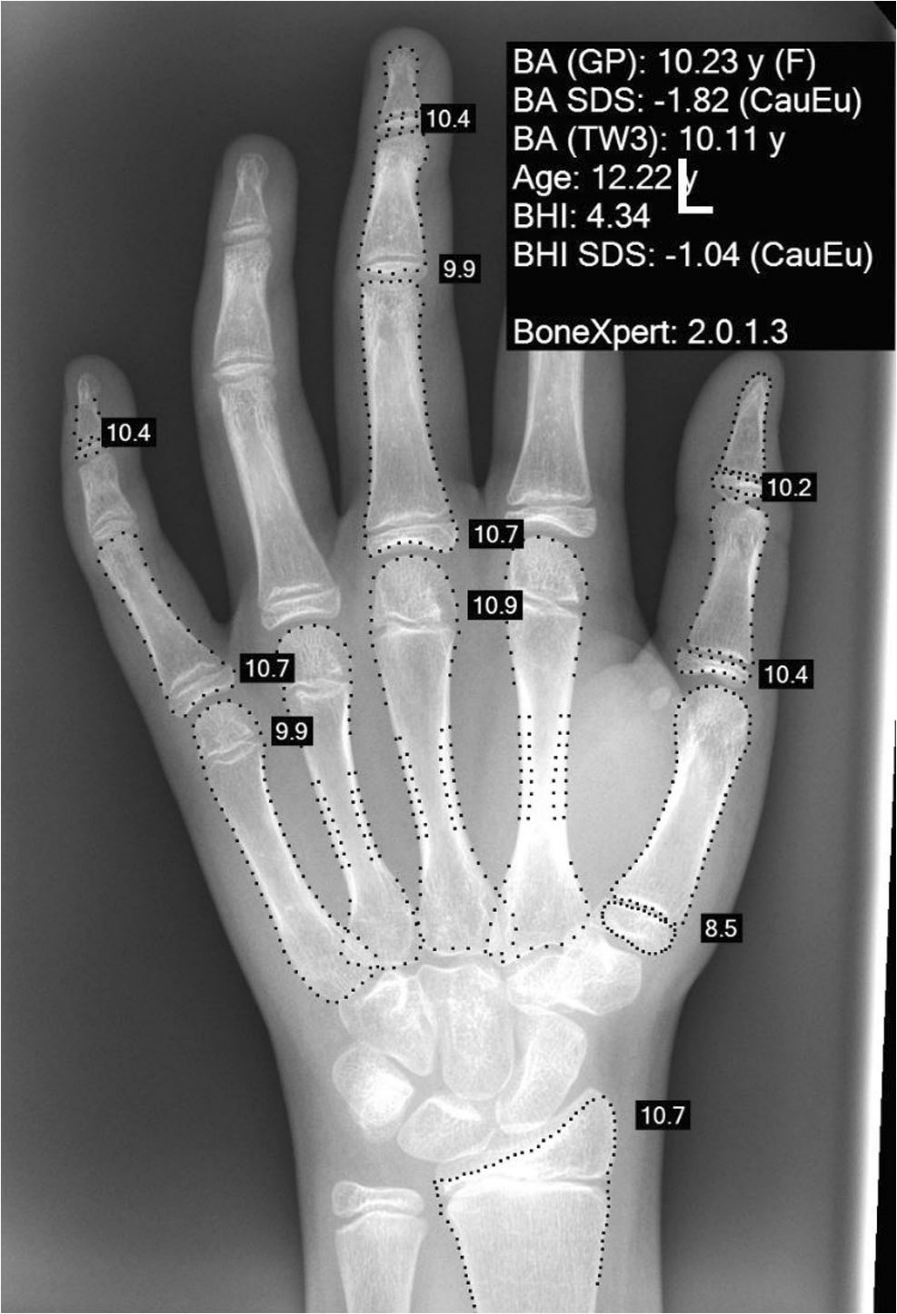
BoneXpert reading of the left hand radiograph of a 12-year-old female. BA (GP), Greulich and Pyle bone age; SDS, standard deviation score; CauEu, Caucasian, European; TW3; Tanner and Whitehouse 3; BHI, bone health index

In contrast to the G&P atlas, we found that TW3 significantly underestimated females’ ages after 3 years of age. The mean difference between BA and CA was statistically significant in females, especially at the ages of 8, 11, 12 (Fig. 4) and 15 years, for all ethnicities and for Caucasians alone. In Caucasian males, the mean BA was significantly lower than CA at age of 9, 12 and 13 years.

A large number of children included in this study (55%) were of low socioeconomic status according to IMD and socioeconomic status explained 17.8% of the difference between bone age (TW3 method) and chronological age. Although there have been improvements in standard of living over the past decade [16] (expected to advance bone age), our results show delayed BA in girls when using the TW3 method. In line with our results, other studies have shown delayed BA compared with CA in females after the age of 10 years [14, 29, 37]. These results potentially support recent views of some researchers, who argue that the improved secular trend has eased or stopped [38, 39]. As a result of an improving secular trend in standard of living, the TW3 method was established in 2001 such that the TW3 BA is about a year ahead of the previous (TW2) method, especially after the age of 10 or 11 years [8]. Our results suggest that a return to TW2 may be necessary.

Several authors argue that socioeconomic status is the predominant reason behind the difference in skeletal maturational rates among populations [12, 14, 31]. Schmeling et al found that bone age was retarded among 27 studies that reported the socioeconomic status of their participants [12]. This retardation was due to the high socioeconomic status of the children recruited to develop the G&P atlas compared with the children within these studies, such that even the secular trend of increasing standard of living was not sufficient to eliminate any differences in socioeconomic status of the various cohorts [29].

In spite of the likely effects of socioeconomic status, the impact of ethnicity cannot be neglected. Studies on two different ethnic groups residing in the same region have shown that bone age assessment methods may reveal different results [24, 34]. Ontell et al showed that the G&P atlas is applicable to Caucasian girls at all ages but not to boys before the age of 13, while in Asians in the same region, the G&P atlas is applicable to girls at all ages but only to boys between 7 and 13.3 years. Zhang et al concluded that Asian children mature sooner than do Caucasian children, especially between 10 and 13 years of age in girls and between 11 and 15 years of age in boys. In a recent meta-analysis, bone age was significantly delayed in African females, while advanced in Asian males when compared with the G&P standard [40]. Furthermore, it has been shown that young Asian adults reach the end of maturity prior to the age observed through the TW3 method (25–27). Research focusing on South African individuals found that TW3 underestimated CA for boys but not for girls [41]. These variations within populations must be considered when assessing bone age [42]. In this current study, we demonstrated no significant difference between all ethnic groups compared with Caucasians alone; it should be noted that Asians and Africans made up only 20% and 5% of the study population respectively.

Measuring BA according to a subjective technique has a greater likelihood of introducing rating variations across analysts, due to varying degrees of expertise. However, this disadvantage was overcome in the current study through the use of BoneXpert, which is an automated bone age analysis software tool that eliminates observer variability and has the advantage of saving significant time. Our observed 5-month persistent discrepancy between chronological age and TW3 bone age as determined by BoneXpert in females appears to be a disadvantage not of the software per se, but of the reference standard (TW3) on which the software depends. Despite this, the software showed acceptable accuracy when using the G&P and TW3 methods for both sexes with the SEE being approximately ± 1 year.

The limitations of this study include the following:

1. The fact that we did not review hospital notes to ascertain full health in the children (although radiology and ED notes were scrutinised);
2. The exclusion of certain age groups, namely those under 2 years old in females, those under 2.5 years in males and individuals of both sexes aged 15 years or older. In order to save time and eliminate subjectivity, this pragmatic study was performed using BoneXpert; however, this software tool is unable to read images from younger age groups due to limited ossification or non-ossification of epiphyses, while its dependability is questionable when used on older age groups [43];
3. Height and weight and pubertal stage of recruited children were not recorded; it is said that that body mass index affects the rate of skeletal maturation [19, 20]; the prevalence of overweight and obese children is well documented to be rising [44] and should be considered in prospective studies of bone age assessment;
4. We do not know the precise socioeconomic status of the reference children, although those recruited for G&P were said to have “good” socioeconomic status;
5. We used self-reported ethnicity; non-Caucasians were a minority in the current study, yet some researchers have shown that ethnicity is more accurately self-reported in groups other than Caucasian [45–47]; and finally,
6. This study did not set out to be and should not be regarded as a validation study of BoneXpert, since the mean absolute and root mean squared errors were not calculated. Rather, we aimed to correlate G&P and TW3 against known CA of a healthy modern population and found that G&P remains reliable (consistent with the results of a recent systematic review) [48]. The question of accuracy of BoneXpert has already been answered in primary research studies [49–51], whereas as far as we are aware, the assessment of the applicability of the standards themselves has not been previously performed using objective software and only a few have considered socioeconomic status [12, 14, 52–54]. Contrary to our results, these studies have shown delayed bone age in children of low socioeconomic status—it is possible that the degree of deprivation in the children from these studies was greater than in ours.

Progress in medicine, education, industry and economic growth have all contributed to higher socioeconomic status which in turn is expected to have had a positive impact on children’s skeletal maturation [8, 24]. Our results show retardation of BA appears counterintuitive, but may not be if the socioeconomic status of the TW3 reference children was on average higher than that of the children we recruited and suggest that perhaps we should revert to the TW2 method.

## Conclusion

Our results indicate that (1) secular change does not appear to have advanced skeletal maturity of UK children; (2) no significant difference exists between BoneXpert-derived BA and CA when using the G&P atlas; therefore, this method can be utilised for the modern UK population; and (3) BoneXpert-derived TW3 BA in current UK children is consistently below the CA of females by an average of 5 months; the clinical significance of this will have to be determined by the requesting clinician and will be greater in younger children who have a lower standard deviation. Developers of BoneXpert may wish to consider this in future upgrades of the software.

## Electronic supplementary material

Supplementary Table 1 (DOCX 20 kb)

## Abbreviations

BA: Bone age
CA: Chronological age
G&P: Greulich and Pyle
IMD: Index of Multiple Deprivation
TW: Tanner and Whitehouse
UK: United Kingdom
