# Supplementary material for: Applicability of two commonly used bone age assessment methods to twenty-first century UK children
Source: Eur Radiol. 2019 Aug 1;30(1):504–13. doi: 10.1007/s00330-019-06300-x (PMC6890594; doi:10.1007/s00330-019-06300-x)
Supplement: Supplementary file 1 — (DOCX 20 kb) [file 330_2019_6300_MOESM1_ESM.docx]

**Supplementary Table 1:** Mean difference between BA and CA in studies that assessed the reliability of the G&P atlas in Caucasian Children

| **Study** | **Origin/ ethnicity** | **Age (years)** | **N** | **Mean BA-CA (years)** |
| --- | --- | --- | --- | --- |
| **G&P** | | | | |
| Loder et al, 1993 [22] | White | 0-18 | M= 203  F= 177 | M= -0.1  F= 0.07 |
| Ontell et al, 1996 [23] | White | 3-18 | M= 208  F= 130 | M= -0.29  F= 0.14 |
| Buken et al, 2009 [9] | Turkish | 11-16 | M = 169  F = 164 | M = -0.02  F = -0.65 |
| Zhang et al, 2009 [13] | White | 0-18 | M = 164  F = 163 | M = 0.01  F = -0.15 |
| Calfee et al, 2010 [24] | Caucasian | 12-18 | M= 62  F= 76 | M= 0.98  F= 0.66 |
| Santoro et al, 2012 [14] | Italian | 7-15 | M = 243  F = 261 | M = -0.1  F = 0.40 |
| Suri et al, 2012 [19] | White | 9-18 | M = 311  F = 261 | M = 0.50  F = 0.50 |
| Paxton et al, 2013 [15] | Australian | 0-18 | M = 276  F = 130 | M = -0.12  F = -0.30 |
| Hackman & Black 2013 [20] | Scottish | 1-20 | M = 249  F = 157 | M = -0.13  F = -0.16 |
| Mansourvar et al, 2014 [16] | White | 10-16 | M = 46 | M = 0.04 |
| Gungor et al, 2015 [25] | Turkish | 10-18 | M = 259  F = 276 | M = 0.64  F = -0.98 |
| Zabet et al, 2015 [21] | French | 10-19 | M = 100  F = 90 | M = -0.19  F = -0.53 |
| Maggio et al, 2016 [17] | Western Australian | 0-25 | M = 180  F = 180 | M = 0.24  F = -0.14 |
| **TW3** | | | | |
| Buken et al, 2009 [9] | Turkish | 11-16 | M = 169  F = 164 | M = -0.18  F = -0.21 |
| Schmidt et al, 2008 [18] | Germany | 1-18 | M = 48  F = 40 | M = 0.61  F = 0.23 |

M = male

F = female
